# Crystal structure and Hirshfeld surface analysis of 10-([2,3′-bi­thio­phen]-5′-yl)-5,5-di­fluoro-5*H*-4λ^4^,5λ^4^-di­pyrrolo­[1,2-*c*:2′,1′-*f*][1,3,2]di­aza­borinine

**DOI:** 10.1107/S205698902600647X

**Published:** 2026-06-26

**Authors:** Darya K. Polyanskaya, Zlata A. Polianskaia, Victor N. Khrustalev, Mehmet Akkurt, Gizachew Mulugeta Manahelohe, Khudayar I. Hasanov, Narmina A. Guliyeva

**Affiliations:** a RUDN University, 6 Miklukho-Maklaya St., Moscow 117198, Russian Federation; b Zelinsky Institute of Organic Chemistry of RAS, Leninsky Prospect 47, Moscow 119991, Russian Federation; c Department of Physics, Faculty of Sciences, Erciyes University, 38039 Kayseri, Türkiye; d Department of Chemistry, University of Gondar, PO Box 196, Gondar, Ethiopia; e Azerbaijan Medical University, Scientific Research Centre (SRC), A. Kasumzade St. 14, AZ 1022, Baku, Azerbaijan; f Department of Chemical Engineering, Baku Engineering University, Hasan Aliyev str. 120, Khirdalan, Absheron AZ0101, Azerbaijan; Universität Greifswald, Germany

**Keywords:** crystal structure, thio­phene, S-hetero­cycle, disorder, Hirshfeld surface analysis

## Abstract

In the title compound, C_17_H_11_BF_2_N_2_S_2_, the mol­ecular conformation is consolidated by an intra­molecular C—H⋯S inter­action forming an *S*(6) ring. In the crystal, mol­ecules are linked by C—H⋯F inter­actions, forming a three-dimensional network. The terminal thio­phene ring is disordered in a 0.659 (3):0.341 (3) ratio around the C—C bond that connects the thio­phene rings, with a rotation of approximately 180° around the ring. According to a Hirshfeld surface analysis, H⋯H (34.8%), C⋯H/H⋯C (22.1%) and F⋯H/H⋯F (18.6%) inter­actions are the main contributors to the crystal packing.

## Abstract 2

The mol­ecular conformation of the title compound is consolidated by intra­molecular C—H⋯S inter­actions, which form an *S*(6) ring. The mol­ecules are linked in the crystal by C—H⋯F inter­actions into a three-dimensional network.

## 1. Chemical context

BODIPY, 4,4-di­fluoro-4-bora-3a,4a-di­aza-*s*-indacene, and its derivatives are well known for their properties as fluoro­phores. First synthesized in 1968, whereas the core scaffold was isolated and described only in 2009, these compounds represent a prominent class of functional compounds with favorable photophysical properties, including a large molar absorption coefficient, narrow absorption and emission bands, high fluorescence quantum yield, and excellent photochemical stability (Treibs & Kreuzer, 1968 ▸; Schmitt *et al.*, 2009 ▸; Yadav & Misra, 2023 ▸). Owing to these characteristics, they have found widespread applications as fluorescent probes, in cell imaging, as organic light-emitting diodes (OLEDs), dye-sensitized solar cells (DSCs) and in phototherapy (Gai *et al.*, 2023 ▸; Gawale *et al.*, 2024 ▸; Mao *et al.*, 2023 ▸). In particular, BODIPYs have been shown to be promising photosensitizers for photodynamic therapy (PDT), despite certain drawbacks such as absorption at wavelengths below 600 nm, hydro­phobicity, and poor tissue penetration (Zhang *et al.*, 2021 ▸). The structural versatility of BODIPYs, including modifications at specific positions of the core, enables fine-tuning of their chemical and photophysical properties (*e.g*., singlet-oxygen generation, emission wavelength, and fluorescence efficiency), thereby enhancing their photodynamic efficacy, biocompatibility, and overall role in imaging and therapeutic applications in PDT (Prieto-Montero *et al.*, 2020 ▸; Malacarne *et al.*, 2022 ▸). Thus, the photophysical behavior of BODIPY may be governed by the substituent at the *meso*-position, yet replacing the typical six-membered aryl ring with five-membered heterocycles (*e.g*., furan, thio­phene, pyrrole, seleno­phene) has received limited attention. For example, the insertion of a thio­phene ring into this position, followed by modification with a nitro­genous base and the creation of a nucleotide based on it, makes it possible to effectively use the resulting BODIPY scaffold as a fluorescent DNA probe to study bacterial metabolism (Šoltysová *et al.*, 2025 ▸). Therefore, studying the introduction of various heterocycles, especially thio­phene derivatives, into the *meso*-position remains relevant. Herein, we report the synthesis of a BODIPY derivative functionalized with a thio­phene ring at the *meso*-position to investigate its influence on the structural, electronic, and photophysical properties of the resultant fluoro­phore. Previously, we described a two-stage method for obtaining BODIPY-type structures, where various heterocyclic aldehydes were utilized as starting compounds (Sadikhova *et al.*, 2024 ▸; Polianskaia *et al.*, 2026 ▸). This strategy was also applied in this study: [2,3′-bi­thio­phene]-5′-carbaldehyde was taken as the starting mol­ecule and introduced into a condensation reaction with pyrrole under acid catalysis in inert atmosphere. The resulting inter­mediate dipyrrolmethane **1** was then oxidized with DDQ in CH_2_Cl_2_ (30 min), followed by neutralization with DIPEA and subsequent treatment with BF_3_·OEt_2_, providing the corresponding BODIPY complex. The target *meso*-thienyl-substituted BODIPY **2** was isolated in 58% yield after silica gel column chromatography.
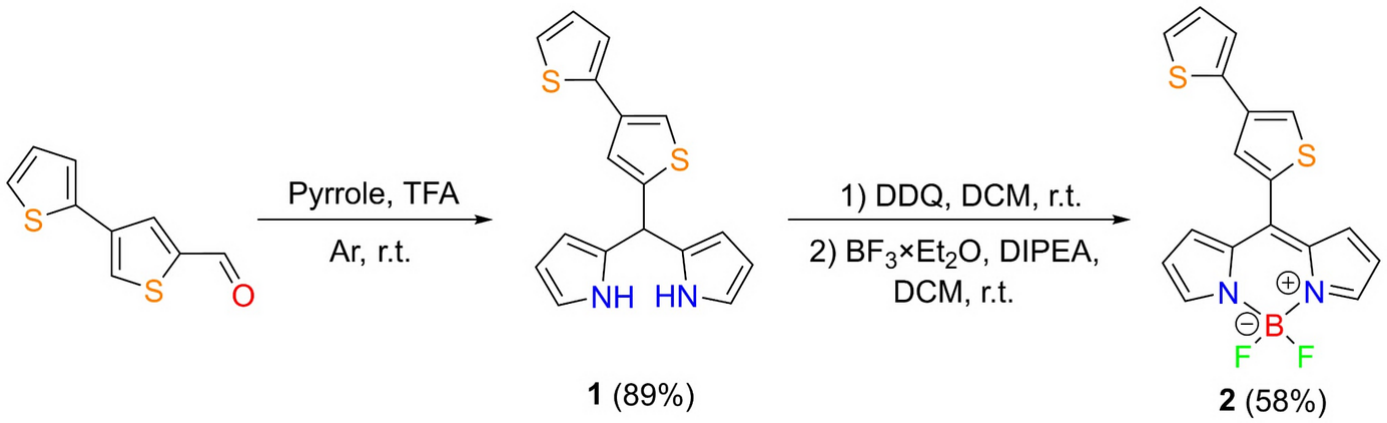


## 2. Structural commentary

**Figure 1:**
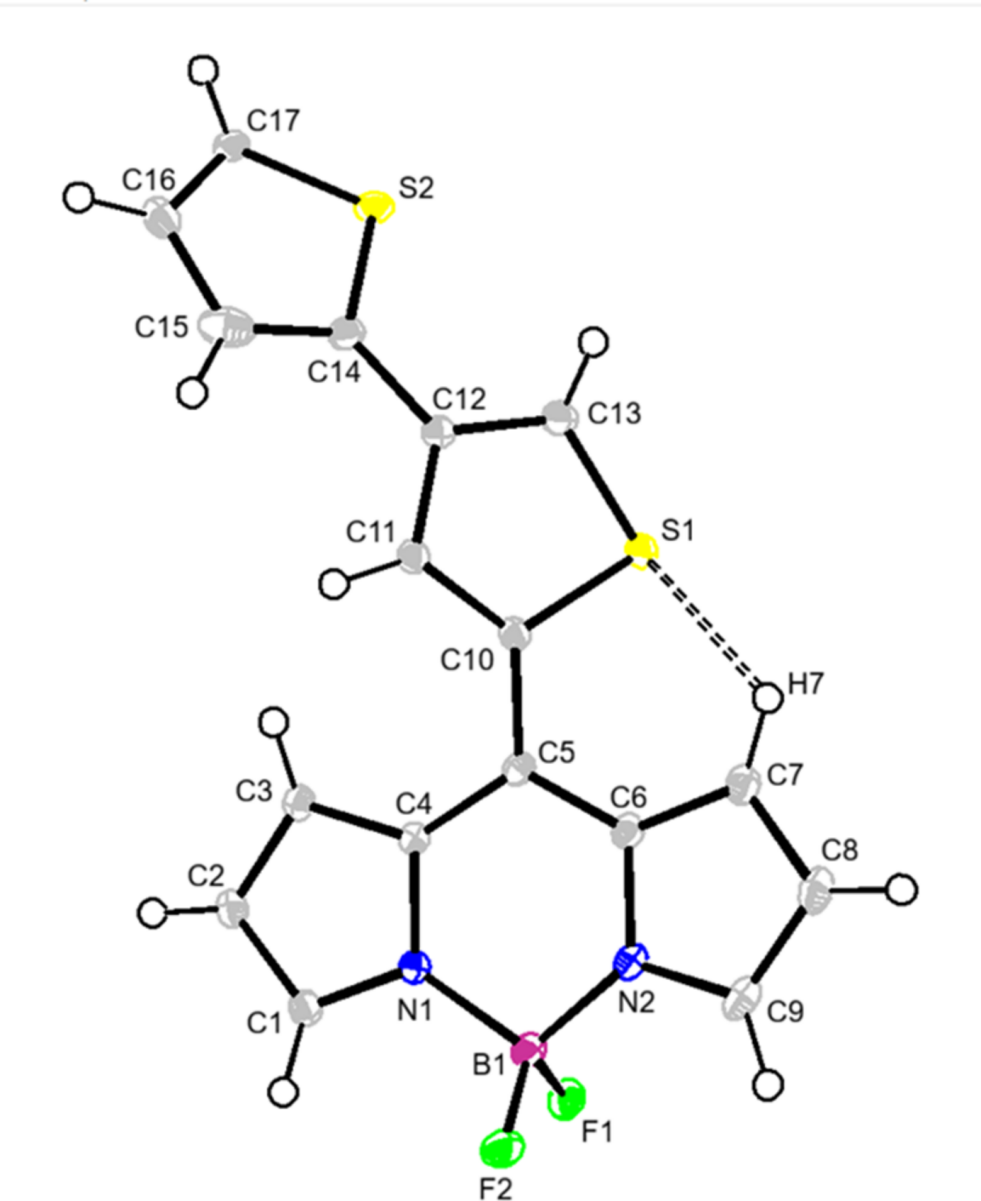
Mol­ecular structure of **2** showing the atomic labelling. Displacement ellipsoids are drawn at the 50% probability level. The intra­molecular hydrogen bond is shown as a dashed line.

The mean plane of the twelve-membered ring system (C1–C9/N1/B1/N2 with an r.m.s. deviation of fitted atoms of 0.0819 Å) makes a dihedral angle of 37.3 (1)°, with the thio­phene ring (S1/C10–C13), while the angles subtended the major and minor components (S2/C14–C17 and S2′/C14/C15′–C17′) of the terminal thio­phene ring and the twelve-membered ring system are 50.9 (3) and 48.8 (6)°, respectively (Fig. 1 ▸). The mol­ecular conformation is consolidated by an intra­molecular C7—H7⋯S1 hydrogen bond, forming an *S*(6) motif (Fig. 1 ▸, Table 1 ▸; Bernstein *et al.*, 1995 ▸). The BODIPY torsion angles F1—B1—N1—C4 and F2—B1—N2—C6 are −103.5 (2) and −135.5 (2)°, respectively. All geometric parameters are normal and consistent with those of related compounds discussed in the *Database survey*.

## 3. Supra­molecular features and Hirshfeld surface analysis

**Figure 2:**
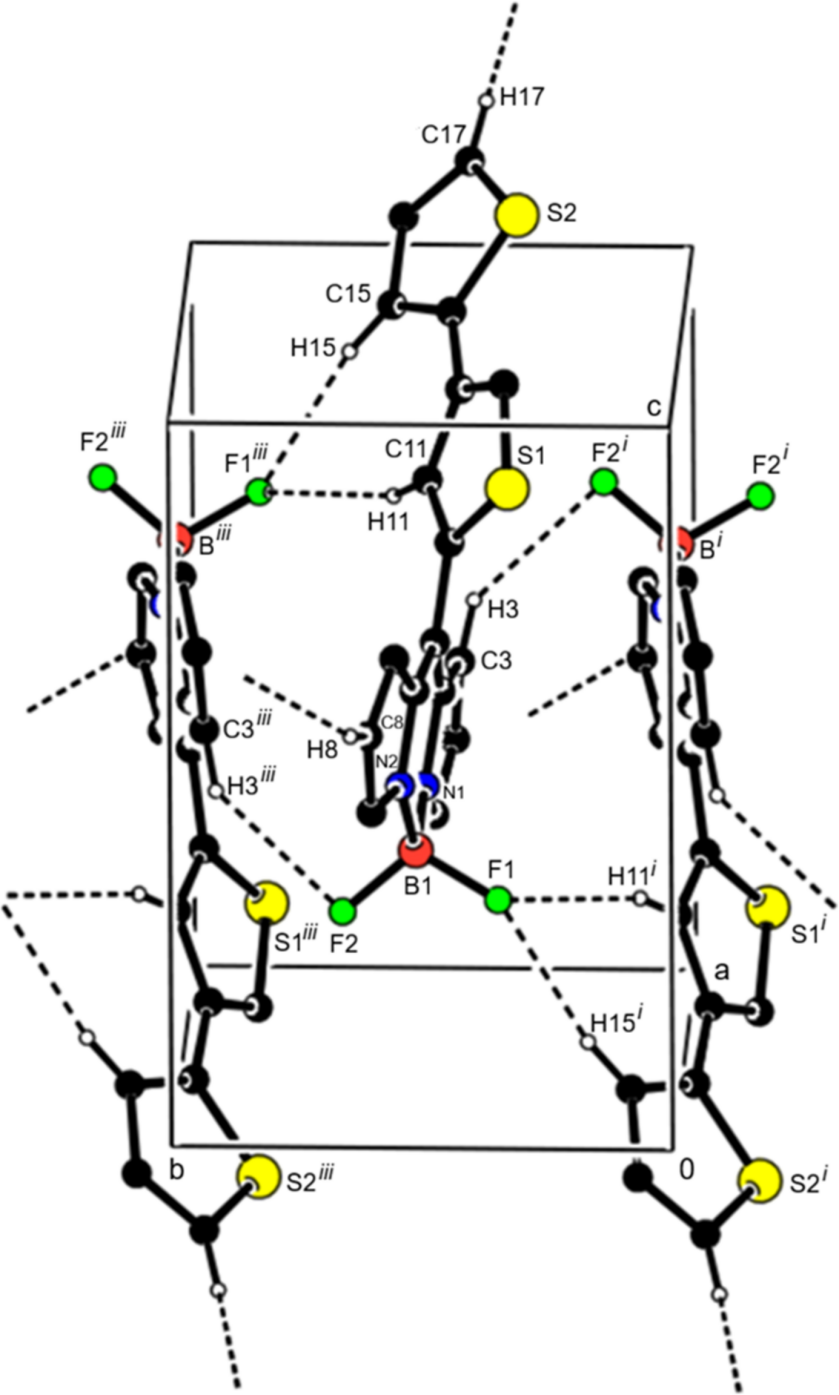
A general view of the C—H⋯F inter­actions (dashed lines) in the unit cell. For symmetry codes, see Table 1. ▸.

**Figure 3:**
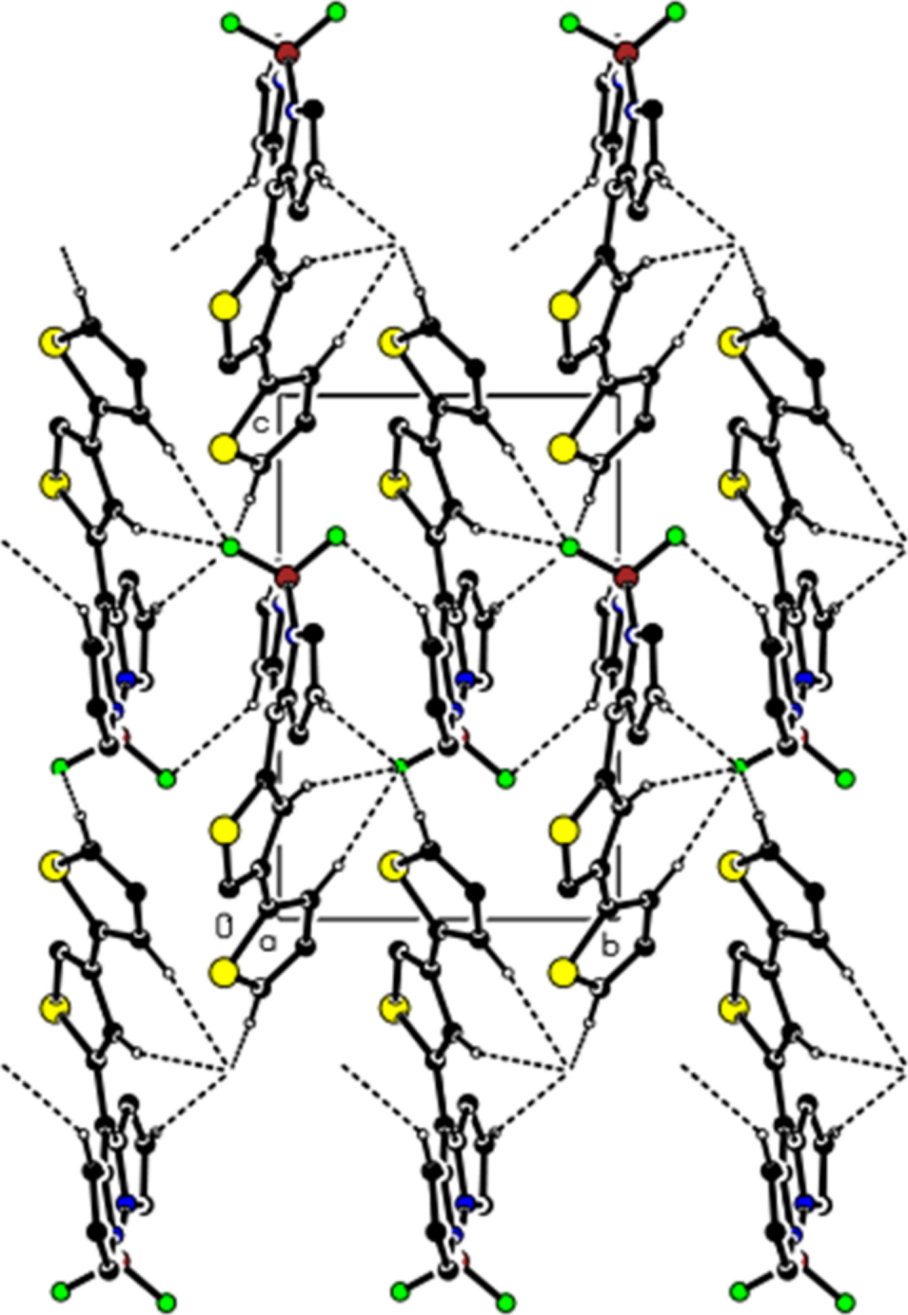
Crystal packing viewed along the *a* axis showing the C—H⋯F inter­actions (dashed lines). H atoms not involved in hydrogen bonding are omitted.

In the crystal, mol­ecules are linked by C—H⋯F inter­actions, forming a three-dimensional framework (Table 1 ▸). A detailed overview of the C—H⋯F inter­actions within the unit cell is given in Fig. 2 ▸. Crystal packing views along the *a* and *c* axes are shown in Figs. 3 ▸ and 4 ▸, respectively. C—H⋯π and π-π inter­actions are not observed.

**Figure 5:**
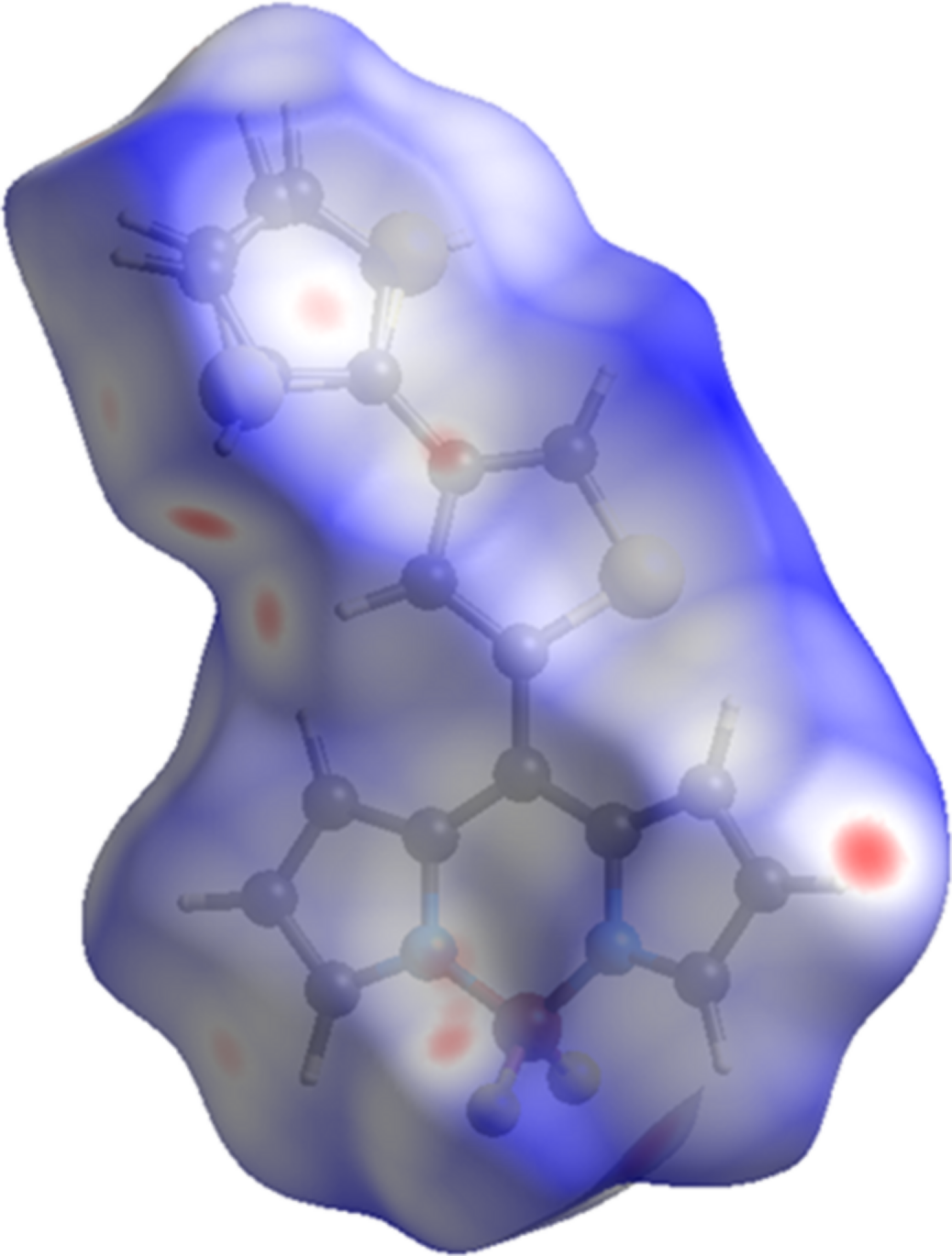
Three-dimensional Hirshfeld surface mapped over *d*_norm_.

The Hirshfeld surface and associated two-dimensional fingerprint plots for the title compound were calculated employing established procedures in *CrystalExplorer17.5* (Spackman *et al.*, 2021 ▸) to determine the influence of weak inter­molecular inter­actions on the mol­ecular packing. The Hirshfeld surfaces mapped over *d*_norm_ using a fixed colour scale of −0.24 (red) to 1.22 (blue) a.u. are shown in Fig. 5 ▸. The few red spots indicate inter­molecular contacts involved in inter­actions (Tables 1 ▸ and 2 ▸).

**Figure 6:**
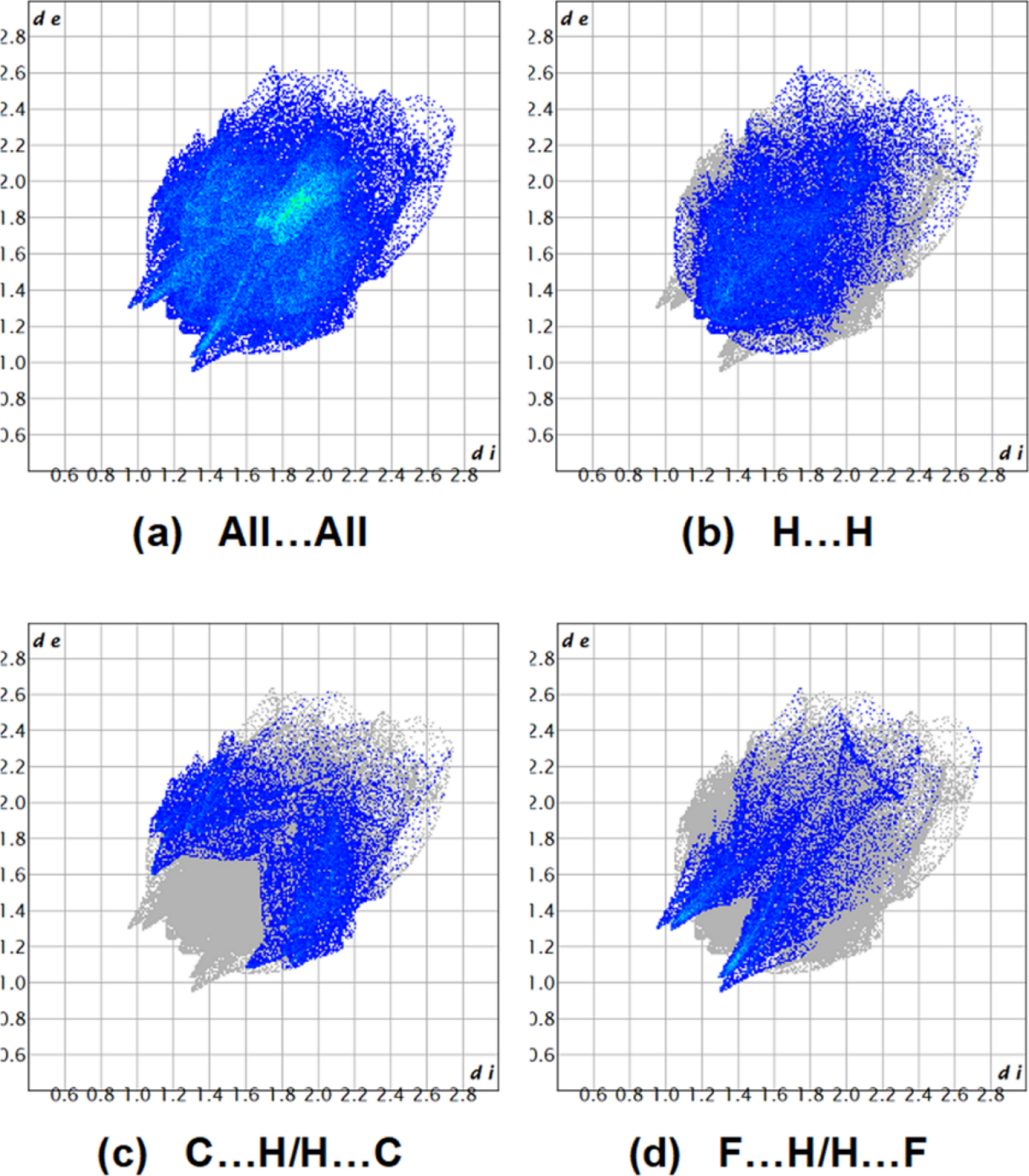
The two-dimensional fingerprint plots showing (*a*) all inter­actions, and those delineated into (*b*) H⋯H, (*c*) C⋯H/H⋯C and (*d*) F⋯H/H⋯F inter­actions. The *d*_i_ and *d*_e_ values are the closest inter­nal and external distances (in Å) from given points on the Hirshfeld surface.

**Figure 7:**
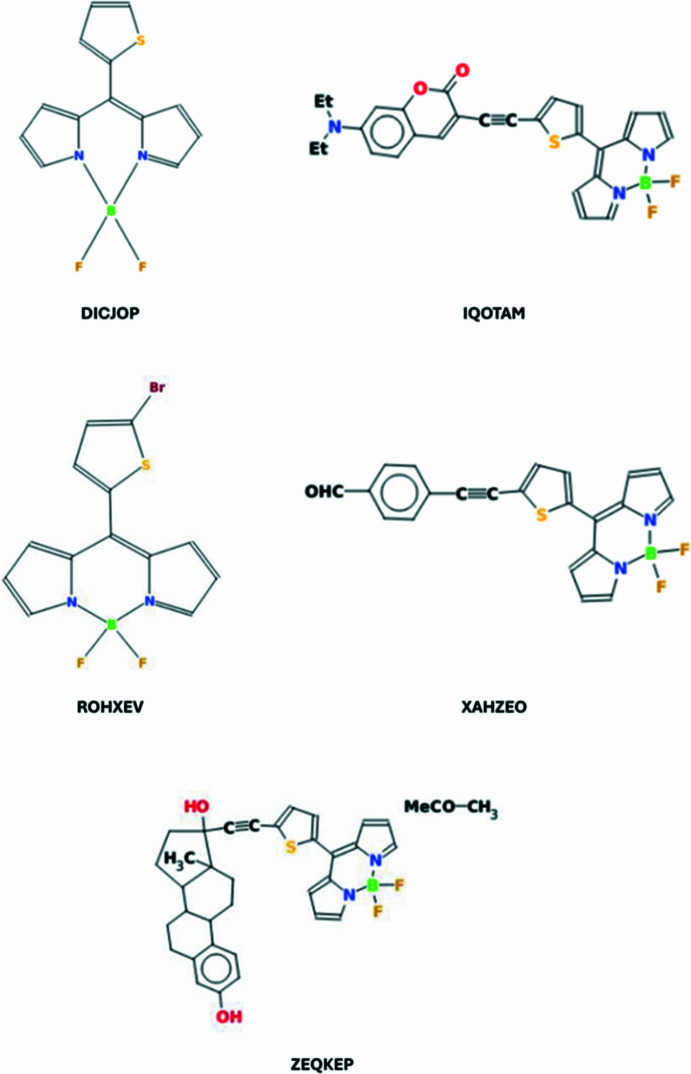
Chemical formulae of compounds DICJOP, IQOTAM, ROHXEV, XAHZEO and ZEQKEP.

Fig. 6 ▸ shows the full two-dimensional fingerprint plot and those delineated into H⋯H (34.8%), C⋯H/H⋯C (22.1%) and F⋯H/H⋯F (18.6%) contacts. The most important inter­action is H⋯H, contributing 34.8% to the overall crystal packing, which is reflected in Fig. 6 ▸*b* as widely scattered points of high density due to the large hydrogen content of the mol­ecule, with small split tips at *d*_e_ ≃ *d*_i_ = 1.25 Å. The pair of characteristic wings in the fingerprint plot arising from C⋯H/H⋯C contacts, Fig. *7c*, has a 22.1% contribution to the Hirshfeld surface with the tips at *d*_e_ + *d*_i_ = 2.70 Å. The F⋯H/H⋯F inter­actions have a 18.6% contribution to the Hirshfeld surface with a pair of sharp spikes characteristic of quite strong inter­actions and *d*_e_ + *d*_i_ ≃ 2.25 Å (Fig. 6 ▸*d*). Other contacts with smaller contributions to the Hirshfeld surface have a less significant effect on the crystal packing: S⋯H/H⋯S (7.1%), C⋯C (6.9%), S⋯C/C⋯S (3.5%), N⋯H/H⋯N (3.3%), F⋯C/C⋯F (2.3%), N⋯C/C⋯N (0.9%), S⋯S (0.3%) and F⋯S/S⋯F (0.2%).

## 4. Database survey

A search of the Cambridge Structural Database (CSD, version 6.00, updated April 2025; Groom *et al.*, 2016 ▸) for a thio­phene-substituted BODIPY revealed five compounds: CSD refcodes DICJOP (Choi *et al.*, 2007 ▸), IQOTAM (Ordóñez-Hernández *et al.*, 2021 ▸), ROHXEV (Farfán–Paredes *et al.*, 2023 ▸), XAHZEO (Xochitiotzi-Flores *et al.*, 2016 ▸), and ZEQKEP (Martínez-Bourget *et al.*, 2022 ▸); when substitutions on pyrrole were allowed, thirty-four compounds were found (Fig. 7 ▸).

DICJOP crystallizes in the ortho­rhom­bic *P*2_1_2_1_2_1_ space group. IQOTAM and ZEQKEP crystallize in the monoclinic space group *P*2_1_, while ROHXEV and XAHZEO crystallize in the triclinic *P*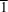 space group.

In DICJOP, C—H⋯F inter­actions link mol­ecules to form layers parallel to the *ac* plane (010) with an 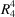
(26) motif protruding along the crystallographic *c* axis and an *R^2^*_2_(14) motif expanding the layer into the *a* direction. In IQOTAM, there are two independent mol­ecules in the asymmetric unit, which are inversion conformers. They form an extensive three-dimensional network with C—H⋯F plus C—H⋯O, C—H⋯S, C—H⋯. and π–π inter­actions. In ROHXEV, the mol­ecules are linked along the *a*-axis direction by C—H⋯F inter­actions, forming *C*(8) zigzag chains. These chains are extended into ribbons by bidirectional C—H⋯F hydrogen bonds forming *R^2^*_2_(10) motifs. The chains are linked along the *b*-axis direction by what may be considered weak C—H⋯π inter­actions, forming layers parallel to the *ab* plane. In XAHZEO, a bidirectional C—H⋯F hydrogen-bonding inter­action of one fluorine with one adjacent mol­ecule forms an *R^2^*_2_(10) ring motif. The second F atom of the BODIPY moiety forms the same hydrogen-bonding motif to the next mol­ecule in the *a*-axis direction. The inter­actions therefore result in broad ribbons extending along the *a*-axis direction. The ribbons are linked by C—H⋯O bonds involving an aldehyde function to form layers in the (012) plane. In ZEQKEP, C—H⋯F inter­actions form chains along the *ac* diagonal. C—H⋯O bonds running parallel to the crystallographic *b* axis link the ribbons into zigzag layers somewhat coplanar with the (401) plane.

In conclusion, the observation of C—H⋯F inter­actions in all of these structures suggests that this inter­action may be generally important in mol­ecular packaging regulation, in particular, for BODIPY derivatives.

## 5. Synthesis and crystallization

The BODIPY synthesis procedure was reported previously (Sadikhova *et al.*, 2024 ▸; Polianskaia *et al.*, 2026 ▸). The starting [2,3′-bi­thio­phene]-5′-carbaldehyde (0.45 g, 2.00 mmol) and pyrrole (3.89 g, 58.00 mmol) were placed into a two-neck flask. The reaction mixture was purged with argon for 10 min. Tri­fluoro­acetic acid (TFA, 26.0 mg, 0.20 mmol) was added dropwise to the reaction under stirring at r.t. After that, the reaction mixture was stirred for an hour under argon. Then Et_3_N (50 µL) was added to pH ∼7. The reaction mixture was poured into water (50 mL) and extracted with ethyl acetate (3 × 10 mL). The target product was purified by column chromatography (eluent: hepta­ne–ethyl acetate 10:1, TLC: hepta­ne/ethyl acetate 4:1); greyish green powdery crystals, yield 89%, 550 mg (1.77 mmol). ^1^H NMR (700.2 MHz, CDCl_3_) (*J*, Hz): δ 8.06 (*br.s*, 2H, NH), 7.27 (*d*, *J* = 1.4 Hz, 1 H, H-2′ Thien), 7.19 (*dd*, *J* = 5.0, 0.9 Hz, 1 H, H-5 Thien), 7.14 (*dd*, *J* = 3.6, 0.9 Hz, 1 H, H-3 Thien), 7.13 (*br.s*, 1 H, H-4′ Thien), 7.02 (*dd*, *J* = 5.0, 3.6 Hz, 1 H, H-4 Thien), 6.75–6.73 (*m*, 2 H, H-5,5′ Pyr), 6.19 (*m*, 2 H, H-4,4′ Pyr), 6.10 (*br.s*, 2 H, H-3,3′ Pyr), 5.75 (*s*, 1 H, CH). ^13^C NMR (176.1 MHz, CDCl_3_): δ 146.8, 139.1, 135.2, 131.5 (2 C), 127.6, 124.6, 123.8, 123.1, 118.7, 117.6 (2 C), 108.6 (2 C), 107.2 (2 C), 39.3. Dipyrrolmethane **1** (542 mg, 1.7 mmol) was dissolved in dry dichloromethane (DCM, 30 ml), after 2,3-di­chloro-5,6-di­cyano­benzo­quinone (DDQ, 1.21 g, 5.3 mmol) was added; the reaction mixture was stirred for 30 min (TLC control), poured into water (80 mL) and extracted with DCM (3 × 30 mL). The organic layer was dried with anhydrous Na_2_SO_4_, concentrated *in vacuo* and the residue was dissolved in dry DCM (20 ml) without further purification. Boron trifluoride etherate (4.5 ml, 34.9 mmol) and an equal volume of diiso­propyl­ethyl­amine (DIPEA, 4.5 ml) were added. The solution was stirred under room temperature for 1 h (TLC control) and then poured into water (80 mL), extracted with DCM (3 × 30 mL) and washed with saturated Na_2_CO_3_ (3 × 30 mL). The organic layer was dried with anhydrous Na_2_SO_4_, the target product **2** was purified by column chromatography (eluent: ethyl acetate/hexane 1:10); dark-red crystals, yield 58%, 330 mg (0.92 mmol), m.p. 431–432 K. Single crystals of the title compound were grown using the mixed solvents ethyl acetate–hexane at 281 K. ^1^H NMR (700.2 MHz, CDCl_3_) (*J*, Hz): δ 7.96 (*s*, 2 H, H-5,5′ Pyr), 7.73 (*dd*, *J* = 7.6, 1.4 Hz, 2 H, H-4,4′ Pyr), 7.34–7.28 (*m*, 4 H, H-2′,3,4′,5 Thien), 7.10 (*dd*, *J* = 5.2, 3.6 Hz, 1 H, H-4 Thien), 6.61 (*m*, 2 H, H-3,3′ Pyr). ^13^C NMR (176.1 MHz, CDCl_3_): δ 144.2 (2 C), 138.8, 137.5, 136.8, 135.2, 134.2, 131.4 (2 C), 131.2, 128.0, 125.0 (2 C), 124.9, 124.2, 118.7 (2 C). ^19^F NMR (658.8 MHz, CDCl_3_): δ −144.8–−145.5 (*m*, 2 F). MS (ESI) *m*/*z*: [*M*]^+^ 356.

## 6. Refinement

Crystal data, data collection and structure refinement details are summarized in Table 3 ▸. All C-bound hydrogen atoms were positioned geometrically (C—H = 0.95 Å) and refined using a riding model, with *U*_iso_(H) = 1.2 *U*_eq_(C). The terminal thio­phene ring (S2/C14–C17) is disordered by a 180° rotation over two orientations around the C12—C14 bond in a 0.659 (3):0.341 (3) ratio. The geometries of the disordered components were restrained to be similar (SAME in *SHELXL*). The rigid bond and similar displacement parameter restraints (DELU and SIMU, respectively) were applied for the atoms involved. One outlier reflection (001), affected by the incident beam-stop, was omitted in the last cycles of the refinement.

## Supplementary Material

Crystal structure: contains datablock(s) I. DOI: 10.1107/S205698902600647X/yz2081sup1.cif

Structure factors: contains datablock(s) I. DOI: 10.1107/S205698902600647X/yz2081Isup2.hkl

Supporting information file. DOI: 10.1107/S205698902600647X/yz2081Isup3.cml

CCDC reference: 2563746

Additional supporting information:  crystallographic information; 3D view; checkCIF report

## Figures and Tables

**Figure 4:**
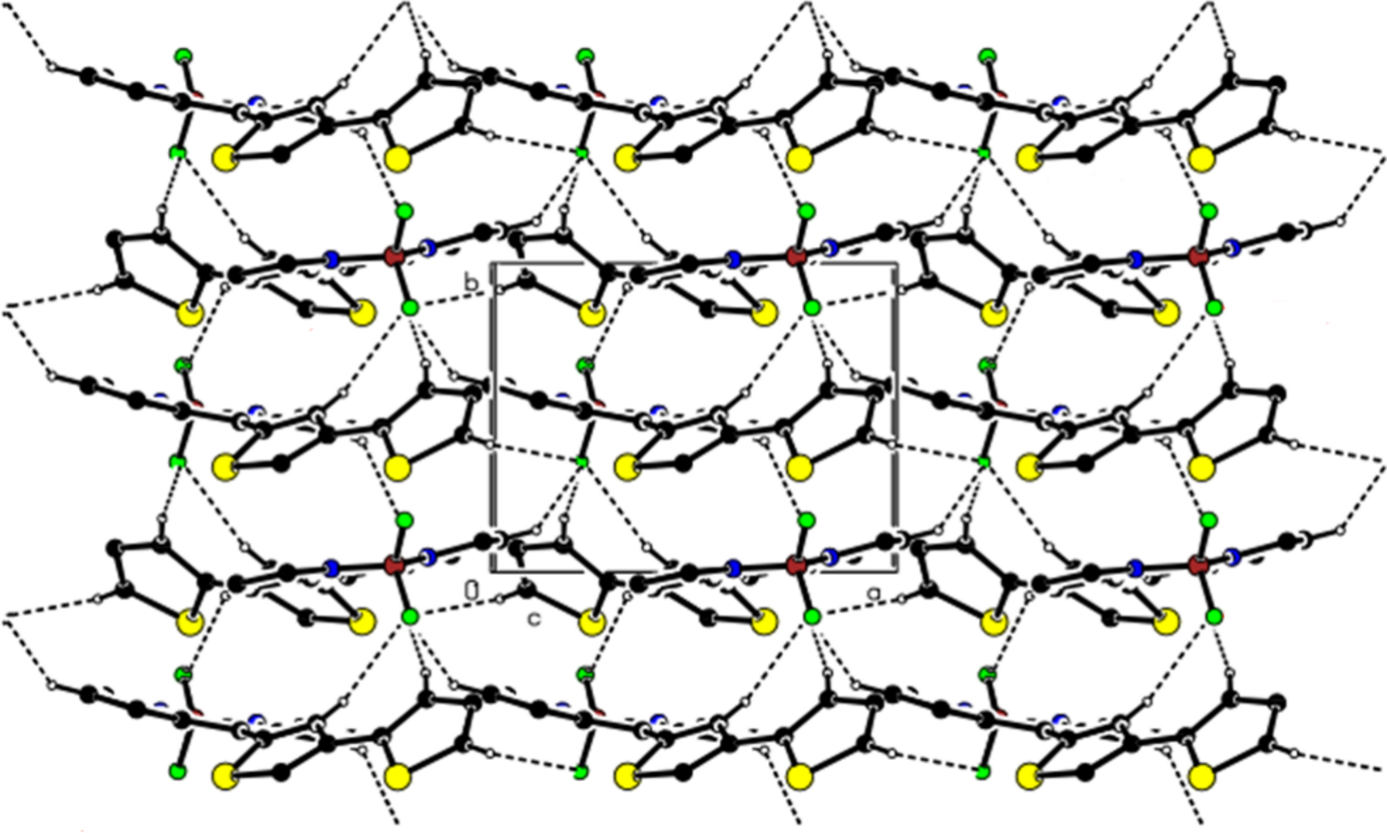
Crystal packing viewed along the *c* axis.

**Table 1:** Hydrogen-bond geometry (Å, °)

*D*—H⋯*A*	*D*—H	H⋯*A*	*D*⋯*A*	*D*—H⋯*A*
C3—H3⋯F2^i^	0.95	2.55	3.362 (2)	144
C7—H7⋯S1	0.95	2.68	3.1895 (19)	114
C8—H8⋯F1^ii^	0.95	2.43	3.229 (2)	142
C11—H11⋯F1^iii^	0.95	2.54	3.4360 (19)	156
C15—H15⋯F1^iii^	0.95	2.49	3.426 (13)	168
C17—H17⋯F1^iv^	0.95	2.38	3.255 (11)	153

Symmetry codes: (i) 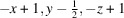; (ii) 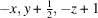; (iii) 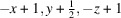; (iv) 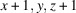.

**Table 2:** Summary of short inter­atomic contacts (Å)

Contact	Distance	Symmetry operation
H7⋯H16*A*^*a*^	2.57	1 − *x*, 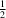 + *y*, 2 − *z*
F1⋯H15^*a*^	2.49	1 − *x*, − 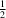 + *y*, 1 − *z*
S2^*a*^⋯H1	3.18	*x*, *y*, 1 + *z*
F1⋯*H17	2.38	−1 + *x*, *y*, −1 + *z*
F1⋯H8	2.43	−*x*, − 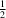 + *y*, 1 − *z*
H2⋯H9	2.56	1 + *x*, *y*, *z*

Note: (*a*) Atom of the minor occupancy disorder component.

**Table 3:** Experimental details

Crystal data	
Chemical formula	C_17_H_11_BF_2_N_2_S_2_
*M*_r_	356.21
Crystal system, space group	Monoclinic, *P*2_1_
Temperature (K)	100
*a*, *b*, *c* (Å)	9.4693 (2), 7.2412 (1), 11.2392 (2)
β (°)	95.512 (1)
*V* (Å^3^)	767.10 (2)
*Z*	2
Radiation type	Mo *K*α
μ (mm^−1^)	0.37
Crystal size (mm)	0.20 × 0.12 × 0.10

Data collection	
Diffractometer	Bruker D8 QUEST PHOTON-III area detector
Absorption correction	Multi-scan (*SADABS*; Krause *et al.*, 2015 ▸)
*T*_min_, *T*_max_	0.674, 0.746
No. of measured, independent and observed [*I* > 2σ(*I*)] reflections	24975, 5559, 5142
*R*_int_	0.041
(sin θ/λ)_max_ (Å^−1^)	0.758

Refinement	
*R*[*F*^2^ > 2σ(*F*^2^)], *wR*(*F*^2^), *S*	0.030, 0.072, 1.03
No. of reflections	5559
No. of parameters	255
No. of restraints	147
H-atom treatment	H-atom parameters constrained
Δρ_max_, Δρ_min_ (e Å^−3^)	0.35, −0.22
Absolute structure	Refined as an inversion twin
Absolute structure parameter	0.29 (6)

Computer programs: *APEX3* (Bruker, 2013 ▸), *SAINT* (Bruker, 2018 ▸), *SHELXS97* (Sheldrick, 2008 ▸), *SHELXL2014* (Sheldrick, 2015 ▸), *ORTEP-3 for Windows* (Farrugia, 2012 ▸) and *PLATON* (Spek, 2020 ▸).

## supplementary crystallographic information

### 10-([2,3'-Bithiophen]-5'-yl)-5,5-difluoro-5H-4λ4,5λ4-dipyrrolo[1,2-c:2',1'-f][1,3,2]diazaborinine Crystal data

**Table:**

C_17_H_11_BF_2_N_2_S_2_	*F*(000) = 364
*M**_r_* = 356.21	*D*_x_ = 1.542 Mg m^−^^3^
Monoclinic, *P*2_1_	Mo *K*α radiation, λ = 0.71073 Å
*a* = 9.4693 (2) Å	Cell parameters from 9989 reflections
*b* = 7.2412 (1) Å	θ = 3.0–32.3°
*c* = 11.2392 (2) Å	µ = 0.37 mm^−^^1^
β = 95.512 (1)°	*T* = 100 K
*V* = 767.10 (2) Å^3^	Prism, red
*Z* = 2	0.20 × 0.12 × 0.10 mm

### 10-([2,3'-Bithiophen]-5'-yl)-5,5-difluoro-5H-4λ4,5λ4-dipyrrolo[1,2-c:2',1'-f][1,3,2]diazaborinine Data collection

**Table:**

Bruker D8 QUEST PHOTON-III area detector diffractometer	5142 reflections with *I* > 2σ(*I*)
Radiation source: fine-focus sealed X-ray tube	*R*_int_ = 0.041
φ and ω scans	θ_max_ = 32.6°, θ_min_ = 2.7°
Absorption correction: multi-scan (SADABS; Krause *et al.*, 2015)	*h* = −14→14
*T*_min_ = 0.674, *T*_max_ = 0.746	*k* = −10→10
24975 measured reflections	*l* = −17→17
5559 independent reflections	

### 10-([2,3'-Bithiophen]-5'-yl)-5,5-difluoro-5H-4λ4,5λ4-dipyrrolo[1,2-c:2',1'-f][1,3,2]diazaborinine Refinement

**Table:**

Refinement on *F*^2^	Secondary atom site location: difference Fourier map
Least-squares matrix: full	Hydrogen site location: inferred from neighbouring sites
*R*[*F*^2^ > 2σ(*F*^2^)] = 0.030	H-atom parameters constrained
*wR*(*F*^2^) = 0.072	*w* = 1/[σ^2^(*F*_o_^2^) + (0.0365*P*)^2^ + 0.1049*P*] where *P* = (*F*_o_^2^ + 2*F*_c_^2^)/3
*S* = 1.03	(Δ/σ)_max_ < 0.001
5559 reflections	Δρ_max_ = 0.35 e Å^−^^3^
255 parameters	Δρ_min_ = −0.22 e Å^−^^3^
147 restraints	Absolute structure: Refined as an inversion twin
Primary atom site location: difference Fourier map	Absolute structure parameter: 0.29 (6)

### 10-([2,3'-Bithiophen]-5'-yl)-5,5-difluoro-5H-4λ4,5λ4-dipyrrolo[1,2-c:2',1'-f][1,3,2]diazaborinine Special details

**Table:**

Geometry. All esds (except the esd in the dihedral angle between two l.s. planes) are estimated using the full covariance matrix. The cell esds are taken into account individually in the estimation of esds in distances, angles and torsion angles; correlations between esds in cell parameters are only used when they are defined by crystal symmetry. An approximate (isotropic) treatment of cell esds is used for estimating esds involving l.s. planes.
Refinement. Refined as a 2-component inversion twin.

### 10-([2,3'-Bithiophen]-5'-yl)-5,5-difluoro-5H-4λ4,5λ4-dipyrrolo[1,2-c:2',1'-f][1,3,2]diazaborinine Fractional atomic coordinates and isotropic or equivalent isotropic displacement parameters (Å^2^)

**Table:**

	*x*	*y*	*z*	*U*_iso_*/*U*_eq_	Occ. (<1)
S1	0.33169 (4)	0.33970 (7)	0.83032 (3)	0.01449 (9)	
C14	0.71857 (18)	0.4686 (3)	0.97857 (15)	0.0151 (3)	
S2	0.7570 (2)	0.3378 (3)	1.1014 (2)	0.0156 (3)	0.659 (3)
C15	0.8301 (13)	0.5897 (19)	0.9628 (11)	0.027 (2)	0.659 (3)
H15	0.828588	0.673475	0.897655	0.032*	0.659 (3)
C16	0.9441 (13)	0.5772 (19)	1.0512 (11)	0.0196 (13)	0.659 (3)
H16	1.027020	0.651489	1.054491	0.024*	0.659 (3)
C17	0.9198 (11)	0.445 (2)	1.1313 (11)	0.0156 (14)	0.659 (3)
H17	0.985589	0.413450	1.197436	0.019*	0.659 (3)
S2'	0.8456 (6)	0.6140 (8)	0.9467 (5)	0.0186 (7)	0.341 (3)
C15'	0.768 (2)	0.368 (3)	1.0892 (19)	0.021 (3)	0.341 (3)
H15A	0.714098	0.273369	1.121888	0.026*	0.341 (3)
C16'	0.899 (2)	0.425 (4)	1.141 (2)	0.017 (3)	0.341 (3)
H16A	0.944480	0.381311	1.215129	0.020*	0.341 (3)
C17'	0.953 (3)	0.551 (4)	1.071 (2)	0.022 (3)	0.341 (3)
H17A	1.044587	0.602867	1.088825	0.027*	0.341 (3)
F1	0.21820 (11)	0.35646 (18)	0.28958 (9)	0.0187 (2)	
F2	0.23232 (12)	0.66759 (18)	0.26818 (11)	0.0211 (2)	
N1	0.41373 (15)	0.5112 (2)	0.39744 (12)	0.0127 (3)	
N2	0.17288 (15)	0.5490 (2)	0.45742 (13)	0.0138 (3)	
C1	0.52374 (18)	0.4979 (3)	0.33101 (16)	0.0154 (3)	
H1	0.517554	0.510899	0.246561	0.018*	
C2	0.64899 (19)	0.4620 (3)	0.40412 (16)	0.0154 (3)	
H2	0.741425	0.449050	0.378963	0.019*	
C3	0.61206 (17)	0.4491 (3)	0.52039 (16)	0.0138 (3)	
H3	0.674394	0.423098	0.589710	0.017*	
C4	0.46383 (17)	0.4819 (2)	0.51653 (15)	0.0122 (3)	
C5	0.37090 (17)	0.4875 (2)	0.60681 (15)	0.0119 (3)	
C6	0.22474 (18)	0.5219 (2)	0.57619 (15)	0.0131 (3)	
C7	0.11248 (18)	0.5563 (3)	0.64747 (16)	0.0157 (3)	
H7	0.117053	0.548041	0.732113	0.019*	
C8	−0.00633 (19)	0.6045 (3)	0.57061 (18)	0.0184 (4)	
H8	−0.097830	0.634997	0.592717	0.022*	
C9	0.03554 (19)	0.5994 (3)	0.45480 (18)	0.0178 (4)	
H9	−0.024200	0.627299	0.384311	0.021*	
C10	0.42658 (17)	0.4603 (2)	0.73114 (15)	0.0125 (3)	
C11	0.55834 (17)	0.5100 (2)	0.78462 (15)	0.0128 (3)	
H11	0.624807	0.580531	0.745589	0.015*	
C12	0.58456 (18)	0.4450 (2)	0.90406 (15)	0.0135 (3)	
C13	0.46880 (17)	0.3523 (3)	0.93995 (14)	0.0154 (3)	
H13	0.465755	0.301006	1.017451	0.018*	
B1	0.2566 (2)	0.5224 (3)	0.34767 (17)	0.0146 (3)	

### 10-([2,3'-Bithiophen]-5'-yl)-5,5-difluoro-5H-4λ4,5λ4-dipyrrolo[1,2-c:2',1'-f][1,3,2]diazaborinine Atomic displacement parameters (Å^2^)

**Table:**

	*U*^11^	*U*^22^	*U*^33^	*U*^12^	*U*^13^	*U*^23^
S1	0.01389 (16)	0.01582 (17)	0.01392 (16)	−0.00373 (16)	0.00209 (13)	0.00032 (17)
C14	0.0145 (7)	0.0175 (8)	0.0128 (7)	0.0011 (6)	0.0000 (6)	−0.0020 (6)
S2	0.0171 (5)	0.0170 (8)	0.0122 (5)	0.0015 (5)	−0.0016 (4)	0.0033 (4)
C15	0.038 (4)	0.024 (4)	0.018 (3)	0.004 (3)	−0.001 (2)	0.006 (2)
C16	0.020 (3)	0.022 (3)	0.018 (3)	−0.002 (2)	0.005 (2)	0.004 (2)
C17	0.012 (3)	0.022 (4)	0.012 (3)	0.0026 (19)	−0.002 (2)	0.001 (2)
S2'	0.0174 (9)	0.0240 (15)	0.0129 (13)	−0.0027 (8)	−0.0057 (9)	0.0055 (10)
C15'	0.023 (4)	0.021 (7)	0.021 (6)	−0.006 (4)	0.009 (3)	0.002 (4)
C16'	0.020 (6)	0.018 (5)	0.012 (4)	0.001 (4)	0.006 (4)	0.006 (3)
C17'	0.018 (4)	0.028 (8)	0.019 (7)	0.000 (4)	−0.005 (4)	0.004 (4)
F1	0.0164 (4)	0.0208 (5)	0.0188 (5)	−0.0043 (5)	0.0012 (4)	−0.0067 (5)
F2	0.0189 (5)	0.0240 (6)	0.0193 (5)	−0.0007 (4)	−0.0029 (4)	0.0068 (4)
N1	0.0118 (6)	0.0140 (6)	0.0121 (6)	−0.0017 (5)	0.0010 (5)	0.0003 (5)
N2	0.0108 (6)	0.0146 (6)	0.0156 (6)	0.0000 (5)	−0.0008 (5)	−0.0015 (5)
C1	0.0151 (7)	0.0169 (8)	0.0143 (7)	−0.0023 (6)	0.0026 (6)	−0.0005 (6)
C2	0.0132 (7)	0.0167 (8)	0.0168 (7)	−0.0007 (6)	0.0040 (6)	−0.0003 (6)
C3	0.0116 (7)	0.0137 (7)	0.0161 (7)	0.0001 (6)	0.0010 (6)	0.0006 (6)
C4	0.0109 (6)	0.0134 (7)	0.0121 (7)	−0.0005 (6)	0.0006 (5)	0.0003 (6)
C5	0.0116 (6)	0.0102 (7)	0.0135 (7)	−0.0010 (5)	−0.0004 (5)	−0.0013 (5)
C6	0.0112 (6)	0.0137 (7)	0.0143 (7)	−0.0005 (6)	0.0011 (5)	−0.0027 (6)
C7	0.0128 (7)	0.0166 (8)	0.0178 (7)	−0.0012 (6)	0.0023 (6)	−0.0043 (6)
C8	0.0113 (7)	0.0191 (8)	0.0246 (9)	0.0011 (6)	0.0016 (7)	−0.0057 (7)
C9	0.0116 (7)	0.0180 (8)	0.0231 (9)	0.0013 (6)	−0.0025 (7)	−0.0028 (7)
C10	0.0132 (7)	0.0122 (7)	0.0124 (7)	−0.0013 (6)	0.0026 (5)	0.0001 (6)
C11	0.0126 (7)	0.0129 (7)	0.0130 (7)	−0.0010 (6)	0.0018 (5)	−0.0006 (6)
C12	0.0138 (7)	0.0145 (7)	0.0122 (7)	−0.0007 (6)	0.0011 (6)	−0.0007 (6)
C13	0.0164 (7)	0.0165 (7)	0.0133 (6)	−0.0017 (7)	0.0010 (5)	−0.0001 (7)
B1	0.0134 (8)	0.0158 (8)	0.0142 (8)	−0.0018 (7)	−0.0008 (6)	−0.0006 (7)

### 10-([2,3'-Bithiophen]-5'-yl)-5,5-difluoro-5H-4λ4,5λ4-dipyrrolo[1,2-c:2',1'-f][1,3,2]diazaborinine Geometric parameters (Å, º)

**Table:**

S1—C13	1.7043 (17)	N2—C9	1.348 (2)
S1—C10	1.7334 (17)	N2—C6	1.391 (2)
C14—C15	1.397 (10)	N2—B1	1.541 (2)
C14—C12	1.462 (2)	C1—C2	1.401 (2)
C14—C15'	1.476 (18)	C1—H1	0.9500
C14—S2'	1.664 (5)	C2—C3	1.388 (2)
C14—S2	1.685 (3)	C2—H2	0.9500
S2—C17	1.729 (9)	C3—C4	1.420 (2)
C15—C16	1.398 (15)	C3—H3	0.9500
C15—H15	0.9500	C4—C5	1.406 (2)
C16—C17	1.350 (9)	C5—C6	1.416 (2)
C16—H16	0.9500	C5—C10	1.459 (2)
C17—H17	0.9500	C6—C7	1.413 (2)
S2'—C17'	1.705 (18)	C7—C8	1.395 (3)
C15'—C16'	1.39 (2)	C7—H7	0.9500
C15'—H15A	0.9500	C8—C9	1.397 (3)
C16'—C17'	1.341 (17)	C8—H8	0.9500
C16'—H16A	0.9500	C9—H9	0.9500
C17'—H17A	0.9500	C10—C11	1.380 (2)
F1—B1	1.399 (2)	C11—C12	1.422 (2)
F2—B1	1.385 (2)	C11—H11	0.9500
N1—C1	1.342 (2)	C12—C13	1.378 (2)
N1—C4	1.393 (2)	C13—H13	0.9500
N1—B1	1.541 (2)		
			
C13—S1—C10	91.83 (8)	C2—C3—C4	107.31 (15)
C15—C14—C12	128.8 (5)	C2—C3—H3	126.3
C12—C14—C15'	127.7 (7)	C4—C3—H3	126.3
C12—C14—S2'	123.8 (2)	N1—C4—C5	120.68 (14)
C15'—C14—S2'	108.4 (7)	N1—C4—C3	107.41 (14)
C15—C14—S2	110.5 (5)	C5—C4—C3	131.91 (15)
C12—C14—S2	120.73 (15)	C4—C5—C6	119.64 (15)
C14—S2—C17	91.5 (4)	C4—C5—C10	119.58 (14)
C14—C15—C16	114.2 (9)	C6—C5—C10	120.77 (15)
C14—C15—H15	122.9	N2—C6—C7	107.68 (15)
C16—C15—H15	122.9	N2—C6—C5	120.32 (15)
C17—C16—C15	110.6 (11)	C7—C6—C5	131.62 (16)
C17—C16—H16	124.7	C8—C7—C6	107.38 (16)
C15—C16—H16	124.7	C8—C7—H7	126.3
C16—C17—S2	113.2 (9)	C6—C7—H7	126.3
C16—C17—H17	123.4	C7—C8—C9	106.62 (16)
S2—C17—H17	123.4	C7—C8—H8	126.7
C14—S2'—C17'	92.4 (8)	C9—C8—H8	126.7
C16'—C15'—C14	114.1 (15)	N2—C9—C8	110.27 (17)
C16'—C15'—H15A	122.9	N2—C9—H9	124.9
C14—C15'—H15A	122.9	C8—C9—H9	124.9
C17'—C16'—C15'	109 (2)	C11—C10—C5	127.63 (15)
C17'—C16'—H16A	125.5	C11—C10—S1	110.75 (13)
C15'—C16'—H16A	125.5	C5—C10—S1	121.47 (12)
C16'—C17'—S2'	116 (2)	C10—C11—C12	113.17 (15)
C16'—C17'—H17A	122.1	C10—C11—H11	123.4
S2'—C17'—H17A	122.1	C12—C11—H11	123.4
C1—N1—C4	108.23 (14)	C13—C12—C11	111.46 (15)
C1—N1—B1	125.17 (14)	C13—C12—C14	124.08 (16)
C4—N1—B1	125.80 (14)	C11—C12—C14	124.43 (16)
C9—N2—C6	108.06 (15)	C12—C13—S1	112.74 (13)
C9—N2—B1	125.85 (15)	C12—C13—H13	123.6
C6—N2—B1	126.08 (14)	S1—C13—H13	123.6
N1—C1—C2	110.22 (15)	F2—B1—F1	109.36 (15)
N1—C1—H1	124.9	F2—B1—N1	111.66 (15)
C2—C1—H1	124.9	F1—B1—N1	108.84 (15)
C3—C2—C1	106.81 (15)	F2—B1—N2	110.79 (15)
C3—C2—H2	126.6	F1—B1—N2	110.52 (15)
C1—C2—H2	126.6	N1—B1—N2	105.61 (14)
			
C15—C14—S2—C17	0.2 (9)	C6—C7—C8—C9	−0.2 (2)
C12—C14—S2—C17	179.4 (6)	C6—N2—C9—C8	−0.6 (2)
C12—C14—C15—C16	180.0 (9)	B1—N2—C9—C8	178.19 (17)
S2—C14—C15—C16	−0.9 (14)	C7—C8—C9—N2	0.5 (2)
C14—C15—C16—C17	1.4 (19)	C4—C5—C10—C11	32.3 (3)
C15—C16—C17—S2	−1.2 (18)	C6—C5—C10—C11	−146.84 (18)
C14—S2—C17—C16	0.6 (13)	C4—C5—C10—S1	−142.80 (14)
C12—C14—S2'—C17'	−179.3 (12)	C6—C5—C10—S1	38.0 (2)
C15'—C14—S2'—C17'	−1.2 (17)	C13—S1—C10—C11	−1.23 (14)
C12—C14—C15'—C16'	−178.6 (19)	C13—S1—C10—C5	174.63 (15)
S2'—C14—C15'—C16'	3 (3)	C5—C10—C11—C12	−173.38 (17)
C14—C15'—C16'—C17'	−4 (4)	S1—C10—C11—C12	2.2 (2)
C15'—C16'—C17'—S2'	3 (4)	C10—C11—C12—C13	−2.2 (2)
C14—S2'—C17'—C16'	−1 (3)	C10—C11—C12—C14	176.20 (16)
C4—N1—C1—C2	−0.7 (2)	C15—C14—C12—C13	−165.1 (8)
B1—N1—C1—C2	−170.93 (16)	C15'—C14—C12—C13	15.0 (14)
N1—C1—C2—C3	1.3 (2)	S2'—C14—C12—C13	−167.3 (3)
C1—C2—C3—C4	−1.4 (2)	S2—C14—C12—C13	16.0 (3)
C1—N1—C4—C5	−179.88 (16)	C15—C14—C12—C11	16.7 (8)
B1—N1—C4—C5	−9.8 (3)	C15'—C14—C12—C11	−163.2 (14)
C1—N1—C4—C3	−0.1 (2)	S2'—C14—C12—C11	14.5 (4)
B1—N1—C4—C3	169.99 (16)	S2—C14—C12—C11	−162.26 (19)
C2—C3—C4—N1	0.9 (2)	C11—C12—C13—S1	1.2 (2)
C2—C3—C4—C5	−179.35 (18)	C14—C12—C13—S1	−177.18 (14)
N1—C4—C5—C6	0.4 (2)	C10—S1—C13—C12	−0.02 (16)
C3—C4—C5—C6	−179.24 (18)	C1—N1—B1—F2	−55.8 (2)
N1—C4—C5—C10	−178.71 (15)	C4—N1—B1—F2	135.73 (17)
C3—C4—C5—C10	1.6 (3)	C1—N1—B1—F1	65.0 (2)
C9—N2—C6—C7	0.5 (2)	C4—N1—B1—F1	−103.45 (19)
B1—N2—C6—C7	−178.32 (16)	C1—N1—B1—N2	−176.27 (16)
C9—N2—C6—C5	−173.11 (16)	C4—N1—B1—N2	15.2 (2)
B1—N2—C6—C5	8.1 (3)	C9—N2—B1—F2	45.9 (2)
C4—C5—C6—N2	0.4 (2)	C6—N2—B1—F2	−135.48 (17)
C10—C5—C6—N2	179.52 (16)	C9—N2—B1—F1	−75.5 (2)
C4—C5—C6—C7	−171.47 (19)	C6—N2—B1—F1	103.14 (19)
C10—C5—C6—C7	7.7 (3)	C9—N2—B1—N1	166.97 (17)
N2—C6—C7—C8	−0.2 (2)	C6—N2—B1—N1	−14.4 (2)
C5—C6—C7—C8	172.44 (19)		

### 10-([2,3'-Bithiophen]-5'-yl)-5,5-difluoro-5H-4λ4,5λ4-dipyrrolo[1,2-c:2',1'-f][1,3,2]diazaborinine Hydrogen-bond geometry (Å, º)

**Table:**

*D*—H···*A*	*D*—H	H···*A*	*D*···*A*	*D*—H···*A*
C3—H3···F2^i^	0.95	2.55	3.362 (2)	144
C7—H7···S1	0.95	2.68	3.1895 (19)	114
C8—H8···F1^ii^	0.95	2.43	3.229 (2)	142
C11—H11···F1^iii^	0.95	2.54	3.4360 (19)	156
C15—H15···F1^iii^	0.95	2.49	3.426 (13)	168
C17—H17···F1^iv^	0.95	2.38	3.255 (11)	153

Symmetry codes: (i) −*x*+1, *y*−1/2, −*z*+1; (ii) −*x*, *y*+1/2, −*z*+1; (iii) −*x*+1, *y*+1/2, −*z*+1; (iv) *x*+1, *y*, *z*+1.
